# Global transcriptome analysis of pig induced pluripotent stem cells derived from six and four reprogramming factors

**DOI:** 10.1038/sdata.2019.34

**Published:** 2019-02-26

**Authors:** Tomokazu Fukuda, Koji Doi, Kenichiro Donai, Kouhei Takahashi, Hisato Kobayashi, Takashi Hirano, Katsuhiko Nishimori, Hiroshi Yasue

**Affiliations:** 1 Graduate School of Science and Engineering, Iwate University, 4-3-5 Ueda, Morioka, Iwate, 020-8551 Japan; 2 Soft Path Engineering Research Center (SPERC), Iwate University, 4-3-5 Ueda, Morioka, Iwate, 020-8551 Japan; 3 Tsukuba Gene Technology Laboratories Inc., Tsuchiura, 6-320 Arakawaoki, 300-0873 Japan; 4 Graduate School of Agricultural Science, Tohoku University, 468-1, Aramaki Aza Aoba, Aoba-ku, Sendai 980-8572 Japan; 5 NODAI Genome Research Center, Tokyo University of Agriculture, 1-1-1 Sakuragaoka, Setagaya-ku, Tokyo, 156-8502 Japan; 6 Department of Embryology, Nara Medical University, 840 Shijo-cho, Kashihara, Nara, 634-8521 Japan; 7 Faculty of Agriculture, Tokyo University of Agriculture, 1737 Funako, Atsugi-shi, Kanagawa, 243-0034 Japan

**Keywords:** Induced pluripotent stem cells, Induced pluripotent stem cells, RNA sequencing, Gene expression analysis

## Abstract

Pigs are important, both for agriculture and as animal models for human diseases. However, due to the lack of embryonic stem cells, the possibility of genetic modification is quite limited. To overcome this limitation, induced pluripotent stem (iPS) cells have been derived from pigs. Despite the public availability of a large number of expression datasets from mice, rats, and primates-derived iPS cells, the expression profile of pig-derived iPS cells is quite limited. Furthermore, there is no dataset focused on the profiling of pig-derived iPS cell with six reprogramming factors (Oct3/4, Sox2, Klf4, c-Myc, Lin28, and Nanog). Here, we used Illumina RNA sequencing platform to characterize the mRNA expression of four-factor derived and six-factor derived pig iPS cells. We observed that the expression levels of whole genes in our established six factors derived iPS cells and parent fibroblast, and compared with that of iPS cells with four factors in public database. These data are valuable in understanding species difference in the reprogramming process of stem cells, and could help identify the key regulating genes involved in the process.

## Background & Summary

The progress of next-generation sequencing technology has caused a technological breakthrough at the whole-genome level in a large number of species^1^. Especially, RNA-sequencing (RNA-Seq) has enabled us to take a snapshot of global gene expression in various organs and cells, regardless of any information of a reference genome. RNA-Seq outputs are digital data that can be uploaded to the public database, and sequence information can be shared worldwide.

RNA-Seq analysis also allows us to compare the biological similarity of embryonic stem cells (ES cell) with induced pluripotent stem (iPS) cells. In general, stem cells can be classified into two major subtypes: naïve and primed states^2,3^. ES/iPS cells at naïve state of pluripotency, reflect the characteristics of pre-implantation embryos and are applicable in rodents, which contribute to chimeras and germ line^4,5^. The growth of naïve stem cell depends on the activation of LIF (Leukemia Inhibitory Factor) signaling, whereby the cell forms colonies with three-dimensional shape.

On the other hand, primed cells have the characteristics of post-implantation embryos and rarely contribute to chimeras and germ line. In brief, primed cells are already at a more differentiated stage compared to naïve cells. Primate ES/iPS cells were conventionally believed to be established in primed state^6,7^. However, recent publications have demonstrated a reliable method for transforming human ES cells from primed to naïve state^8,9^. Transcriptome analysis using RNA-Seq played an important role in identifying the cellular characteristics reported in those articles.

In the case of pig iPS cells, the status of the cells–naïve or primed–remains inconclusive since the pluripotent genes have a wide variety of phenotypes. To understand the biological variety of pig iPS cells, multiple datasets of global gene expression profiling would be needed. Although a significant number of reports on the establishment of pig iPS cells have been published^10–20^, expression profiling data in Sequence Read Archive (SRA) database are quite limited. Therefore, detailed biological features of pig iPS cells need to be addressed with whole expression profiling.

In our previous publication, we had reported that pig iPS cell, derived from six reprogramming factors, has more advantageous than that derived from four factors. Especially, the expression of six reprogramming factors was suitable for X chromosome re-activation^21^, which is one of the mile-stone characteristics of naïve-type stem cells. Our previous data using Ion Torrent sequencing also proved that the expression of six reprogramming factors was more advantageous to activate various pluripotent genes. Although the data obtained from Ion Torrent is suggestive, at least 20 M reads would be necessary to obtain a quantitative evaluation of the relatively low-expressing genes. The data obtained in our previous publication seem insufficient in terms of the number of sequencing reads required to conclude. This situation led us to detect the global expression profile of pig iPS cells, derived from the expression of six reprogramming factors, using Illumina short-read sequencer, HiSeq 2500. Currently, there are no publicly available dataset of six factor-derived pig iPS cells using Illumina sequencing platform.

The aim of this study was to clarify the difference of mRNA expression profiles between pig iPS cells derived from six and four reprogramming factors. We found relevant submitted data from two research groups on pig iPS cells with four reprogramming factors, in SRA^22,23^. We could compare ours with these gene expressions since both datasets were obtained with Illumina sequencer. In this study, we describe the detailed expression profile of pig iPS cells derived from four and six reprogramming factors. Multiple analyses demonstrated that the pig iPS cells derived from six factors formed independent clusters compared to those derived from four factors, and were distant from fibroblasts. Furthermore, we detected that the expression levels of various naïve-specific genes were relatively elevated in pig iPS cells derived from six factors. Our data set would contribute to the understanding of biological differences between the iPS cells derived from six and four reprogramming factors, and provide the scientific explanation of how diversity of pluripotency-related genes related to the process of animal evolution.

## Methods

### RNA preparation and sequencing

Pig-derived iPS cells were cultured on the feeder cells derived from mouse embryonic fibroblasts. When iPS cells reached to the confluent condition around 3 × 10^6^ cells/35 mm diameter cell culture dish, the cells were lysed in 700 μl of the RA1 solution of NucleoSpin RNA extraction kit (code: 740955.250, Takara Bio, Shiga, Japan). The total RNAs from pig embryonic fibroblast, iPS clone 1 and iPS clone 2, three RNA samples were submitted to NODAI Genome Research Center for quality control and Next Generation Sequencing. All samples had integrity number (RIN) >9 in Bioanalyzer (Agilent, Santa Clara, CA, USA)^31^. TruSeq RNA libraries were prepared from total RNA according to the manufacturer’s protocol (TruSeq RNA Library Prep Kit v2, Illumina, San Diego, CA, USA). Libraries for the three cDNA samples were sequenced using the Illumina Hiseq 2500 sequencing platform yielding about 45 million 100-bp paired-end sequence reads per sample.

### Quality check and mapping

**Figure 1:**
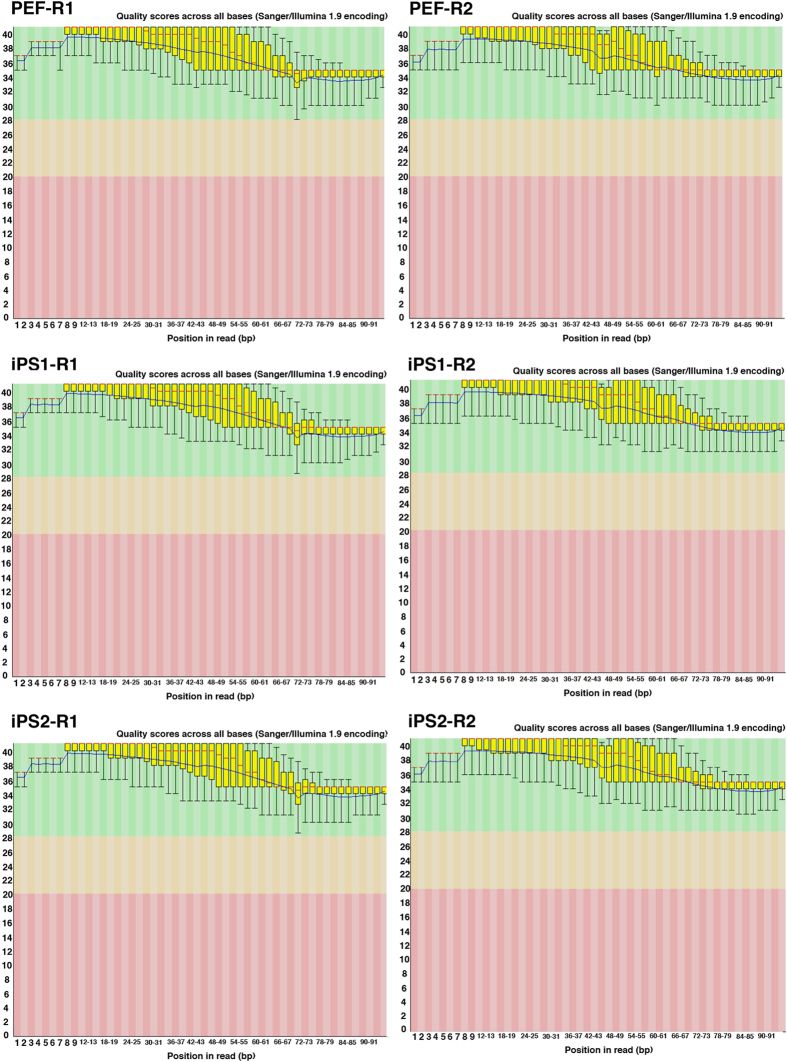
Quality assessment of raw FASTQ files of 100 bp paired-end reads. R1 is first read and R2 is the second read for pair-end sequencing. Box and whisker plots show the quality scores across all bases. The blue line indicates the mean value and the yellow box represents 25–75% ranges. Whisker also represents 10–90% ranges. All plots were automatically generated by FastQC program.

**Figure 2:**
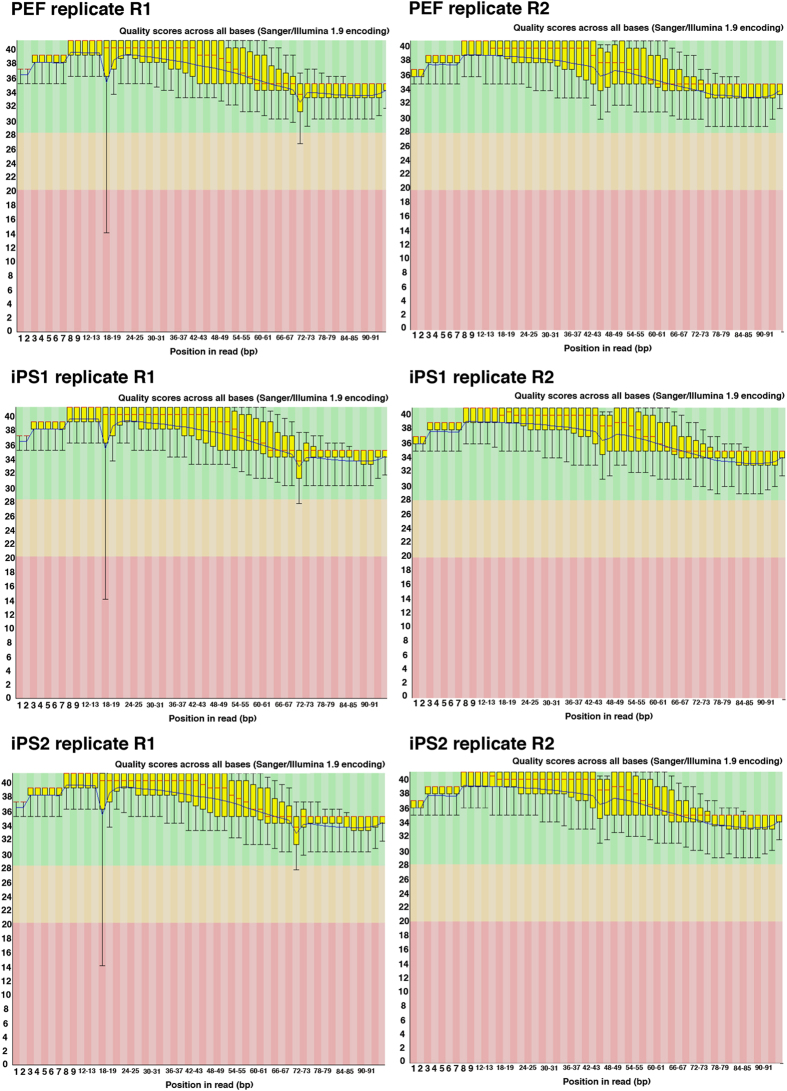
Quality assessment of raw FASTQ files of 100 bp replicated paired-end reads. R1 is first read and R2 is the second read for pair-end sequencing. Box and whisker plots show the quality scores across all bases. The blue line indicates the mean value and the yellow box represents 25–75% ranges. Whisker also represents 10–90% ranges. All plots were automatically generated by FastQC program.

Quality of raw sequencing reads was evaluated using FastQC program. FastQC results plotted the position of nucleotide base call on the x-axis and their corresponding quality score on the y-axis. Figures 1 and 2 shows that all FASTQ sequencing files have mean quality score >36, which conforms with the manufacturer’s standard >33 of our own data. The summary of the data were listed in Table 1. The sample information, such as single read, or paired-end, number of reprogramming factors for pig iPS, were listed in Table 1. We analyzed the 9 data from reference and public data base, and 6 data from our own experiments. The raw read data were recorded in Data Bank of Japan (DDBJ), and have been assigned BioProject accession PRJDB5113 (Data Citation 1).

**Figure 3:**
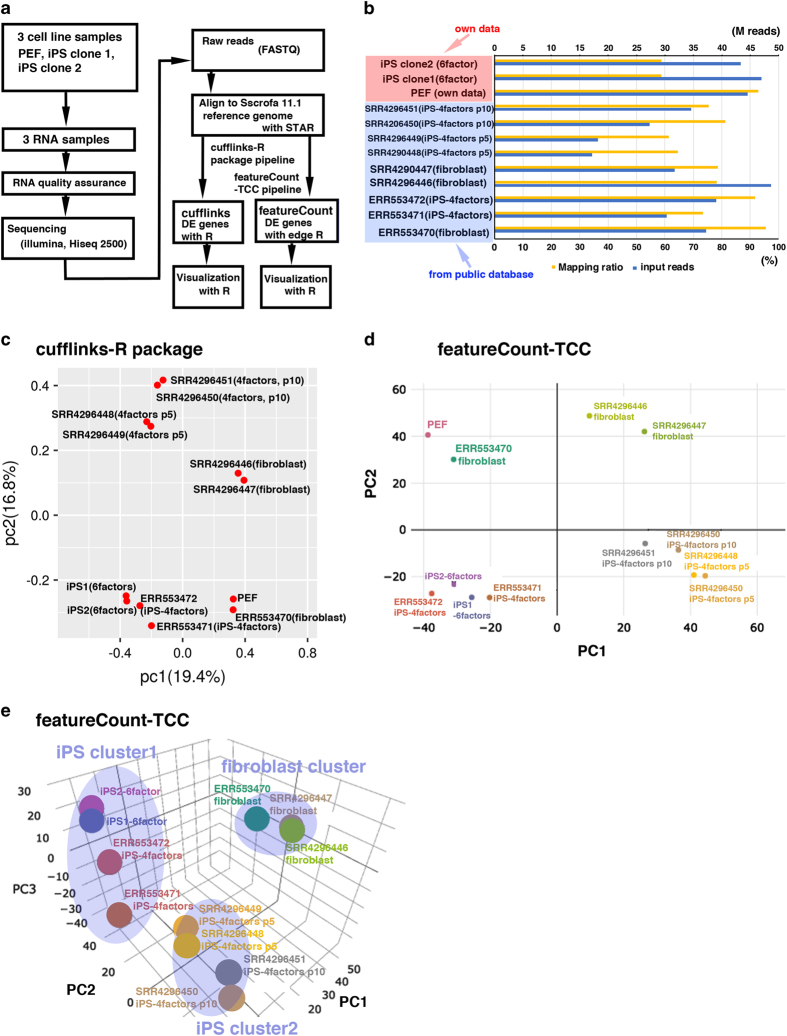
Workflow of the bioinformatics analysis and the results of mapping and following downstream analysis. (**a**) Flowchart of the RNA-Seq and downstream analysis. After the mapping with STAR, we carried out the analysis with two pipelines, cufflinks-R package pipeline, and featureCount-TCC pipeline. (**b**) The blue bar indicates the total number of input reads. The yellow bar represents the mapping rate per sample using STAR. (**c**) Two dimensional PCA analysis with cufflinks-R package pipeline. Similarities among pig fibroblasts and iPS cells were shown as a map. (**d**) Two dimensional PCA analysis with featureCount-TCC pipeline. (**e**) Three dimensional PCA analysis with featureCount-TCC pipeline. The result of the analysis showed three clusters, fibroblast, iPS cluster 1, and iPS cluster2.

Figure 3a shows our experimental workflow following quality validation of the sequence reads. All sequence reads were trimmed using PRINSEQ software for discarding the low-quality reads. In case the adapter sequence remained, cutadapt software was used. The sequencing reads were aligned to the Sus Scrofa Ensembl genome assembly (Sscrofa11.1) using STAR^24^. The rates of uniquely mapped reads ranged from 60–95% (Fig. 2b). After the mapping, the analysis was divided into two pipelines, cufflinks-R package and featureCount-TCC (Fig. 3a).

### Counting gene expression and downstream analysis

For the cufflinks-R package pipeline, the distance matrix was obtained with cufflinks after the alignments. The differentially expressed genes were identified with R package, such as ape (dendrogram analysis), prcomp() in R (PCA analysis), Heatmap.3() in R (heatmap analysis) from results of cufflinks. For the featureCount-TCC analysis, the output with BAM format was processed with featureCount program, and the differentially expressed genes were identified with TCC-GUI program, which provided from website of Dr. Koji Kadota (Tokyo University, Tokyo, Japan) (https://infinityloop.shinyapps.io/TCC-GUI/). The basic algorism of TCC-GUI is TCC (an acronym for Tag Count Comparison)^25^ and normalized gene expression with Edge R^26^. The parameter of TCC-GUI is described in below, normalization method was TMM method, differentially expressed genes were counted with EdgeR, the filtering threshold for low count genes was set at 7, FDR cut off was set as 0.1, elimination of potential differentially expressed genes was set at 0.05. The original code of analysis (two dimensional PCA plot, three dimensional PCA plot, dendrogram analysis, heatmap analysis, expression analysis of identified genes of TCC) were supplied as the function of TCC-GUI (https://infinityloop.shinyapps.io/TCC-GUI/). The output file for the normalized expression level with TCC-GUI (tmm_edger_3_0.1_0.05_TCC_Normalized.csv, Data Citation 2) in Figshare. The list of differentially expressed genes determined with TCC-GUI were also provided in Figshare (tmm_edger_3_0.1_0.05_TCC.csv, Data Citation 2).

### Code availability

The following software and versions were used for quality control, data trimming, and data analysis as described in the main text:

1. FastQC, version 0.11.3 was used for quality check of raw FASTQ sequencing data: http://www.bioinformatics.babraham.ac.uk/projects/fastqc/
2. PRINSEQ, version 0.20.4 was used for trimming sequence data: http://prinseq.sourceforge.net/
3. cutadapt, v1.14 was used for removing adapter sequences: http://cutadapt.readthedocs.io/en/stable/index.html
4. STAR, version 2.6.0c was used for mapping of sequence reads to the pig Sscrofa11.1 genome assembly: https://github.com/alexdobin/STAR/blob/master/doc/STARmanual.pdf
5. Cufflinks, ver 2.2.1 was used to obtain the distance matrix: https://github.com/cole-trapnell-lab/cufflinks
6. R package, Version 3.4.0, https://stat.ethz.ch/pipermail/r-announce/2017/000612.html, featureCount, Version 1.6.3, https://sourceforge.net/projects/subread/files/subread-1.6.3/subread-1.6.3-Linux-x86_64.tar.gz/download
7. TCC-GUI, https://infinityloop.shinyapps.io/TCC-GUI/

## Data Records

Raw FASTQ files for the RNA-Seq were deposited to the DNA Data Bank of Japan (DDBJ), and have been assigned BioProject accession PRJDB5113. The output file for the normalized expression level with TCC-GUI is in Figshare (tmm_edger_3_0.1_0.05_TCC_Normalized.csv, Data Citation 2). The list of differentially expressed genes determined with TCC-GUI were also provided in Figshare (tmm_edger_3_0.1_0.05_TCC.csv, Data Citation 2, 3).

## Technical Validation

### RNA integrity values

Quality of Total RNA was assessed using an Agilent Bioanalyzer to calculate RNA Integrity Number (RIN). The RIN algorithm evaluates the RNA quality in the samples on a scale of one to ten. Illumina sequencing highly recommends RIN >8 for analysis.

### Raw FASTQ file quality

**Figure 4:**
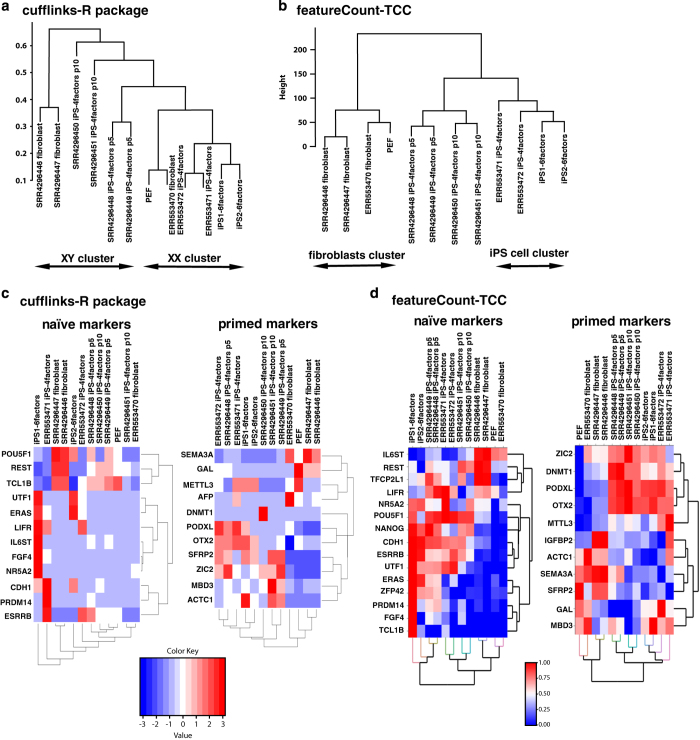
Dendrogram analysis and heatmap analysis of the expression level of naïve and primed-specific genes among fibroblast and multiple pig iPS cell lines. (**a**) Dendrogram analysis with cufflinks-R package pipeline. Result of the analysis forms XY and XX clusters. (**b**) Dendrogram analysis with featureCount-TCC pipeline. Result of the analysis forms fibroblast and iPS clusters. (**c**) Heatmap analysis of naïve and primed specific genes among fibroblast and multiple pig iPS cell lines with cufflinks-R package pipeline. (**d**) Heatmap analysis of naïve and primed specific genes among fibroblast and multiple pig iPS cell lines with featureCount-TCC pipeline.

The results of FastQC showed that the quality score, as the accuracy of base call, was very high (mean > 36) compared to the Illumina standard >33 (Figs 1 and 2). Our sequences were mapped to the Sscrofa 11.1 genome assembly between 60–95% rates (Fig. 3b). After the mapping, analysis pipeline was divided into two lines, cufflinks-R package, and featureCount-TCC analysis. Results of two dimensional PCA plot were shown in Fig 3c and d. Although the two-dimensional PCA plots with both pipelines could not show the difference between fibroblast and pig iPS cells (Fig. 3d), three-dimensional PCA plot with featureCount-TCC pipeline showed the clear unique cluster of fibroblasts, pig iPS cells (Fig. 3e). Furthermore, the dendrogram analysis showed that cufflinks-R package pipeline divided into two groups, XX chromosome samples and XY chromosome samples (Fig. 4a). Interestingly, featureCount-TCC pipeline showed two clusters, fibroblast and pig iPS cells (Fig. 4b).

### Comparison with published studies

**Figure 5:**
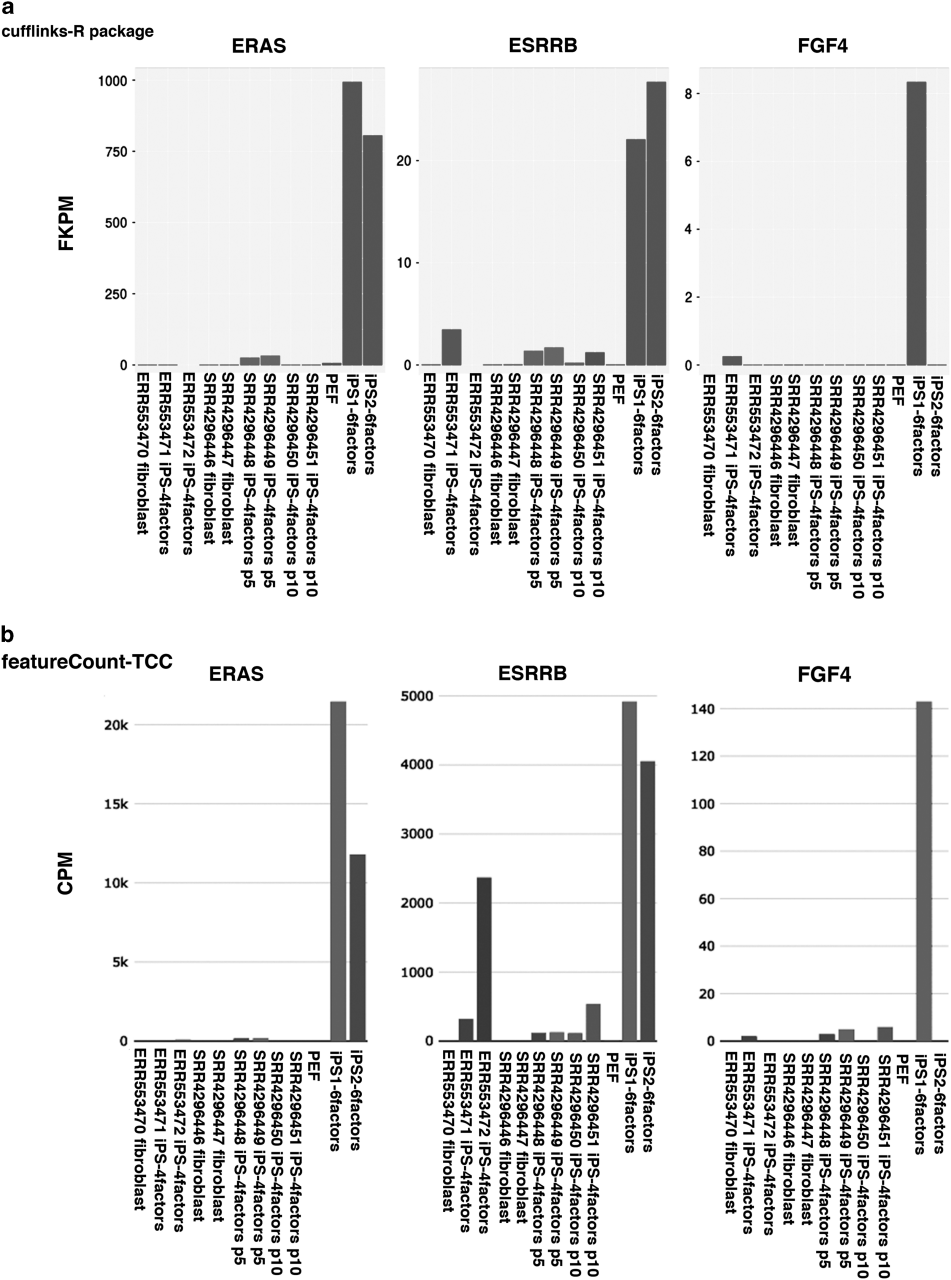
Expression level of naïve specific genes, ERAS, ESRRB, FGF4 among fibroblast and multiple pig iPS cell lines. (**a**) Expression levels with cufflinks-R package pipeline. (**b**) Expression level with featureCount-TCC pipeline.

The current study was compared with two datasets from previous studies. We showed the expression of naïve-specific genes and primed specific genes as a heat map in Fig. 4c,d. As good agreement with dendrogram analysis, the heatmap analysis with featureCount-TCC analysis showed the unique cluster of pig iPS cells with six factor expression, while cufflinks-R package analysis could not form the unique cluster. We focused on three representative genes: *ERAS*, *ESRRB, FDF4* which are well known as naïve markers. These genes had higher expression in our pig iPS cells with six factors than in pig iPS cells with four factors (Fig. 5a,b). These results indicate that pig iPS cells with six factors are located closer to naïve state.

## Usage Notes

We have used Linux OS, Ubuntu 14.04.5 LTS for all analysis.

## Additional information

**How to cite this article**: Fukuda, T. *et al*. Global transcriptome of pig induced pluripotent stem cells derived from six and four reprogramming factors. *Sci. Data*. 6:190034 https://doi.org/10.1038/sdata.2019.34 (2019).

**Publisher’s note**: Springer Nature remains neutral with regard to jurisdictional claims in published maps and institutional affiliations.

## Supplementary Material



## Figures and Tables

**Table 1:** Cellular characteristics of pig fibroblasts and iPS cells in this study.

Accession # or Cell line	Source	Induction method	Cell character	Gender	X-inactivation	Teratoma Formation	Chimerism	Sequence	Reference
ERR553470	fibroblasts	–	–	XX	–	–	–	single	^23^
ERR553471	iPSCs	4 factor	LIF	XX	yes	no data	yes	single	^23^
ERR553472	iPSCs	4 factor	LIF + FGF	XX	–	yes	no data	single	^23^
SRR4296446	fibroblasts	–	–	XY	–	–	–	paired	^22^
SRR4296447	fibroblasts	–	–	XY		–	–	paired	^22^
SRR4296448	iPSCs	4 factor	Rex1 (plus), p5	XY	–	yes	no data	paired	^22^
SRR4296449	iPSCs	4 factor	Rex1 (plus), p5	XY		yes	no data	paired	^22^
SRR4296450	iPSCs	4 factor	Rex1 (−), P10	XY	–	no	no data	paired	^22^
SRR4296451	iPSCs	4 factor	Rex1 (−), P10	XY		no	no data	paired	^22^
PEF, DRX117351, DRX152009	fibroblasts	–	–	XX	–	–	–	paired (2 × 100)	This study
PiPSC1, DRX117349, DRX152010	iPSCs	6 factor	LIF + low molecular inhibitors	XX	yes	yes	not tested	paired (2 × 100)	This study
PiPSC2, DRX117350, DRX152011	iPSCs	6 factor	LIF + low molecular inhibitors	XX	yes	yes	not tested	paired (2 × 100)	This study
In the section of reference, we indicated the origin of the data, from reference or original data.									
